# Kentucky Institution for the Blind

**Published:** 1848-04

**Authors:** 


### KENTUCKY INSTITUTION FOR THE BLIND.

The Sixth Annual Report to the Legislature of Kentucky of the Managers of this valuable School has been sent to us. The number of pupils is 31. During the year four from Indiana have been withdrawn, which we mention for the purpose of adding, that they have been placed in a school at Indianapolis, recently established by that state. We congratulate our young sister (in part a daughter of Kentucky) on this creditable enterprise, in addition to one still more important in its character and bearings, the establishment of an Asylum for the Insane, in the same city; which we understand, will, in the course of the present year, be so far completed as to receive patients.

We learn from the Report before us, that—

"The entire number of pupils received into the Institution, since it was opened for the reception of pupils in 1842, is fifty-three; thirty-two males, and twenty-one females. Of these, only fifteen were born blind; the blindness of thirty-eight was the result of disease or accident."

We cannot but feel and express our surprise, that the number should not have been greater. How slowly inventions for the mere relief of infirmity unaccompanied with pain or danger, make their way among the mass of the people! During the past year, according to the Report, the Director, Mr. Patten, with some of the pupils, visited 15 counties of the State, and "found many blind persons sitting in darkness, ignorance, and helplessness, without hope either for the present or the future state of existence!" That the number in the State is such that the School ought at this period to have more than four times as many as are reported, we cannot doubt. If a more efficient hemp-break had been offered to the people of Kentucky six years ago, it would ere this have spread over the whole State. A man will eagerly adopt a new means of making money, while he reads with indifference, of an establishment for the education of his unfortunate blind child! A sad commentary on human nature. We would again exhort our brethren throughout Kentucky, and in such of the adjoining States as have not established schools for the blind, to exert all the influence in their power, within their respective spheres of action, in making known the existence and advantages of the school, that those who are now vegetating in darkness, may be brought under its beneficial and cheering advantages.

